# Integrating communication dimensions into health models: understanding COVID-19 vaccination through multigroup analysis

**DOI:** 10.3389/fpubh.2025.1609127

**Published:** 2025-06-12

**Authors:** Jimena Collantes-Loo, Edgardo R. Bravo

**Affiliations:** ^1^Faculty of Life Sciences, Universidad de Ciencias Aplicadas, Lima, Peru; ^2^Engineering Department, Universidad Del Pacífico, Lima, Peru

**Keywords:** health communication, health models, trust, vaccination, COVID-19

## Abstract

**Introduction:**

The global COVID-19 pandemic has highlighted the importance of maintaining public engagement with vaccination programs through effective health communication, particularly as initial crisis awareness fades. This study develops and validates an integrated model of vaccination behavior by combining the Theory of Planned Behavior, the Protection Motivation Theory, and the Structural Influence Model of Health Communication with health communication dimensions.

**Methods:**

Using partial least squares analysis of survey data from 320 U.S. adults, the research examines how message components influence cognitive appraisals (threat and response efficacy) and how this relationship is moderated by information-seeking behavior, information processing ease, and trust in government.

**Results:**

Results confirm the core relationships of the Theory of Planned Behavior in vaccination contexts while revealing a temporal evolution in risk appraisals, with severity and susceptibility showing diminished effects on attitudes. While message components show non-significant direct effects on appraisals in the aggregate sample, these relationships become significant among high information seekers and those with greater trust in government. Additionally, vulnerability-focused messages have differential effects based on chronic health status, positively impacting risk perceptions among vulnerable populations while reducing perceived susceptibility in others.

**Conclusion:**

These findings advance the theoretical understanding of health communication and suggest implementing segmented communication strategies based on audience characteristics.

## 1 Introduction

The global impact of the SARS-CoV-2 (also known as COVID-19) pandemic has had significant adverse social, health, and economic effects (1). The annual Gross Domestic Product (GDP) growth rate decreased from 2.8% in 2019 to −3.0% (2). The unemployment rate increased from 5.4% in 2019 to 6.5% in 2020 (3). Furthermore, the global pandemic has severely disrupted health systems, resulting in over 6.8 million deaths (4), placing individuals without resources in a more vulnerable position, and contributing to the deterioration of mental health (5). Furthermore, the mortality rate per 100,000 individuals rose from 715.2 to 835.4 between 2019 and 2020 (6).

The devastating global consequences of COVID-19 underscore the critical importance of developing effective preventive public health strategies. Among these preventive measures, vaccination is one of the most efficacious interventions in reducing the transmission and severity of COVID-19 (7). However, despite its proven effectiveness, vaccine hesitancy has emerged as a significant barrier to comprehensive disease prevention efforts. Vaccine hesitancy is the delay in accepting or refusing vaccines despite the availability of vaccination services (8). Therefore, understanding the reasons for vaccine delay, refusal, and intention is mandatory since convincing the population to accept the vaccine is challenging. During the first year of the pandemic, some countries had a vaccine intention of 27.7%, while others had 93.3% (9). The lowest rates of vaccine intention diminish the probability of achieving herd immunity, resulting in less control of the pandemic.

Among the multiple strategies to deal with vaccine hesitancy, effective health communication has emerged as crucial in shaping public perception and behavioral responses during health crises (10). Research has shown that clear communication with reliable information enhances public understanding and boosts confidence in dealing with pandemics (11, 12). Health communication is vital for vaccination strategy as it influences people’s intentions based on the information they receive (9).

The pivotal role of health communication in influencing vaccination intentions underscores the importance of understanding the theoretical frameworks that explain preventive health behaviors. Recent literature has significantly advanced in explaining them, particularly regarding COVID-19 vaccination. The existing research can be broadly categorized into two main streams: On the one hand, researchers have applied established psychological and health behavior models to explain vaccination decisions. Numerous studies have utilized established theoretical frameworks to explain COVID-19 vaccination behavior (e.g., (13, 14)), providing valuable insights into the cognitive processes and motivational factors underlying vaccination decisions. These include generic socio-cognitive models such as the Theory of Planned Behavior (TPB) (15, 16) and more specific health models like the Protection Motivation Theory (PMT) (17) and the Extended Parallel Process Model (EPPM) (18). Recently, scholars have endeavored to integrate TPB and health models to leverage their complementary aspects and overcome the limitations inherent to each model when considered in isolation. For example, some researchers (19, 20) have incorporated typical risk perceptions into the TPB, thereby addressing the model’s initial lack of health-specific constructs.

On the other hand, more recent studies have begun to incorporate communication factors into explanatory frameworks. Specifically, the Structural Influence Model of Health Communication (SIM-HC) (21, 22) represents an essential advancement in incorporating communication aspects into vaccination studies. SIM-HC provides a macro-level perspective that considers relevant communicational dimensions: information seeking, information processing ease, and trust in the source to explain behavioral constructs predominantly mediated by risk issues (23).

While previous research has brought a valuable advance in comprehending the phenomenon, some research opportunities have been detected. First, a compelling research opportunity emerges from the relative neglect of the message component within the COVID-19 vaccination literature. While health models acknowledge the significance of message components in shaping cognitive appraisals, this construct appears to be underutilized in recent COVID-19 vaccination studies. The existing literature has predominantly employed experimental designs in simulated settings rather than studies in real contexts, focusing on communication design instead of the formation of appraisals (24, 25).

Second, an intriguing research opportunity lies in the potential of integrating message components from health models with the communication dimensions outlined in the SIM-HC literature. While its constructs have been studied separately, their potential interactions remain unexplored. This integrative approach could yield a more holistic understanding of the communication processes underlying vaccination decisions, potentially revealing complex interactions between message content, information processing, and behavioral outcomes.

This study aims to develop and test a model addressing the abovementioned gaps. The proposed model highlights the significance of message components in a real context and their interaction with communication dimensions derived from SIM-HC. In addition, these communication aspects are incorporated into generic social-cognitive models. Specifically, TPB is the pivotal theory around health, and communication models are integrated in a more nuanced and complete picture of the complex interplay between communication factors, cognitive processes, and social influences in shaping vaccination behavior.

The potential implications of this work are extensive, particularly for public health strategy. Findings from this study could inform more effective communication approaches for promoting ongoing vaccination against respiratory pathogens, enabling policymakers and healthcare providers to design and implement more impactful vaccination programs and communication campaigns. This research addresses current gaps in the literature and lays the groundwork for future interdisciplinary studies in public health communication and behavior change.

The paper begins by developing hypotheses that integrate communication dimensions with behavioral models, then outlines the methodology, reports the results, and discusses the implications for public health management and future research.

## 2 Research model development

The integrated model proposed in this study is based on the Theory of Planned Behavior (TPB) and serves as the core framework for understanding this phenomenon. This model extends the TPB by incorporating key elements from risk health literature and health communication research, thereby addressing gaps detected in existing literature. The rest of the section develops the research model, starting with TPB as a pivotal framework.

According to TPB, the model postulates that attitudes and subjective norms primarily influence behavioral intentions. In turn, the perceived benefits and costs shape attitude. This section of the model focuses on the cognitive-social process of shaping vaccination intention.

Risk perceptions (perceived likelihood, severity, and susceptibility) and response efficacy from Protection Motivation Theory (PMT) are incorporated as antecedents of attitude to enrich the TPB framework. This integration captures the cognitive appraisals of health threats and the coping mechanisms individuals engage in vaccination decisions.

To better understand the role of communication in health prevention behaviors, the model proposes that message components (response efficacy, probability of occurrence, and noxiousness) impact their respective cognitive appraisals. Also, it considers that the relationship between the communication of susceptibility and perception of susceptibility is contingent on the individual’s chronic condition.

Finally, the model proposes that communication dimensions (information seeking, information processing ease, and trust in government) moderate the relationship between communication dimensions and their respective cognitive appraisals. The following sections elaborate on each component of the model.

### 2.1 Theory of Planned Behavior (TPB) and vaccination intention

The TPB (15) posits that intentions are the most proximate and robust predictor of actual behavior. They represent an individual’s motivation and preparedness to engage in a particular action. The TPB also proposes that intentions are formed by three determinants: attitude, subjective norms, and perceived behavioral control (PBC). Attitude is the value that an individual ascribes to a given behavior. An individual’s intention to perform a given behavior is strengthened by a more favorable attitude toward that behavior. This is because individuals tend to intend to engage in behaviors perceived as favorable. The subjective norms are the normative expectations of the social group to which the individual belongs. The greater social pressure to engage in a behavior is associated with enhanced intentions. When individuals believe that significant others approve of a given behavior, they are more likely to intend to perform it. The PBC is the individual’s belief in their capacity to control the performance of the behavior in question. PBC is comprised of two key elements: self-efficacy and controllability. Self-efficacy can be defined as an individual’s perception of their capacity to achieve a given behavior successfully. Controllability pertains to an individual’s judgment regarding the availability of resources and opportunities to achieve a given behavior. Higher perceived behavioral control is associated with increased intention. When individuals believe they have the ability and resources to perform a behavior, they are more likely to form intentions. In turn, the TPB posits that an individual’s beliefs about the probable consequences of the behavior, particularly expected benefits and costs, shape the attitude. Finally, the TPB suggests that the relative importance of these relationships can vary depending on the specific behavior and context.

Under the TPB umbrella, this study conceptualizes vaccination attitude as the value of support or hesitancy related to vaccination among the general public. Subjective norms are the perceived social pressure from significant others to get vaccinated. Some empirical studies have supported the relationships between attitude and subjective norms on intention in the context of vaccination in particular (13, 26) and preventive behaviors in general (handwashing and limitation of social contacts) (27). Following the TPB rationale, it is expected that:

*H1:* Attitude positively affects the intention.

*H2:* Subjective norms positively affect the intention.

As previously stated, TPB also considers that beliefs influence attitude formation. In this study, perceived benefits are defined as the belief that vaccination will reduce the threat of disease. In contrast, perceived costs are defined as the belief that vaccination will entail monetary costs or require exertion of effort (19). These constructs are salient aspects in the health prevention literature (28). Even more empirical studies have supported that the perception of benefits and costs influences attitudes toward vaccination (19, 29). Following the tenets of TPB, it is anticipated that:

*H3:* Benefits positively affect attitude.

*H4:* Costs negatively affect attitude.

It should be noted that this study does not consider PBC, as some empirical studies have found a marginal additional impact on vaccination intention. Possibly unlike other behaviors that require ongoing effort or complex skills, vaccination is typically a simple, one-time action that is widely accessible in many contexts. Most individuals may perceive minimal difficulty in obtaining vaccination, resulting in low variability in PBC across the population (13).

### 2.2 Protection Motivation Theory (PMT)—vaccination attitude

While the TPB offers a robust foundation for understanding vaccination behavior, integrating specific constructs from health literature could facilitate a more comprehensive and nuanced understanding of the vaccination phenomenon. The model incorporates risk perceptions and response efficacy, as outlined in Protection Motivation Theory (PMT) (17, 30).

PMT posits that individuals will be self-protective when they perceive a health threat. The Protection Motivation Theory (PMT) posits that two appraisal processes—threat appraisal and coping appraisal—drive the decision to engage in self-protective behaviors.

Threat appraisal depends on the knowledge about the threat that the individual has. Therefore, it is formed by three dimensions of risks. First, perceived likelihood refers to the probability of being harmed by a hazard if no preventive action is taken, as illustrated by the question, “What is the likelihood that you will get the flu this year if you do not get a flu shot?.” Second, perceived susceptibility focuses on individual constitutional vulnerability to an illness, exemplified by questions like “Are you more likely to get the flu than others?” Finally, perceived severity refers to the seriousness of contracting an illness, considering medical and social consequences (e.g., death or impairment in daily activities), such as asking, “If I do not get vaccinated, would my health be seriously endangered? “(31, 32).

The coping appraisal refers to the ability of the individual to cope and avert health threats successfully through self-efficacy, response efficacy, and response cost. This study excludes self-efficacy and costs because the former is part of PCB, and the latter is similar to the TPB construct costs. Perceived response efficacy refers to the belief about how effective the result of preventive behavior, such as getting the vaccine, is in reducing harm.

In general, the PMT suggests that individuals, when faced with a threat to their health, engage in preventive behaviors if they believe that this will reduce their risk or that not performing these behaviors may cause them harm (33).

Based on conceptual and empirical results (19, 34), this study posits that PMT constructs could be conceptualized as beliefs that contribute to shaping the attitude toward vaccination and, therefore, have an indirect effect on intention. Both PMT constructs and TPB beliefs are fundamentally cognitive in nature. They represent mental schemas that individuals use to understand and anticipate the consequences of their actions. This shared cognitive basis reinforces the rationale for incorporating PMT constructs as beliefs within the TPB framework. Also, within TPB, beliefs contribute to attitudes by providing the evaluative information necessary to form an overall judgment about a behavior. Similarly, the PMT constructs provide evaluative information about health threats and protective behaviors, which can directly shape attitudes (35).

Some studies have shown that beliefs about risks may affect attitude, especially in preventive behaviors (26, 36, 37).

Following the TPB tenets and the previous discussion and findings, it is expected that:

*H5:* Response efficacy positively affects attitude.

*H6:* Perceived likelihood positively affects attitude.

*H7:* Perceived severity positively affects attitude.

*H8:* Perceived susceptibility positively affects attitude.

### 2.3 Message components and cognitive appraisals

PMT, in general, and specifically the work of Witte (18), proposes that message components are the critical elements of a fear appeal communication designed to influence cognitive appraisals. These components can be categorized into two main groups: threat components and efficacy components. The first one comprises three types of messages: Communication on the probability of occurrence: Messages that convey information about the likelihood of the threat occurring if no preventive action is taken. Communication on noxiousness: Messages that represent the severity or harm of the threat’s consequences. Communication on the susceptibility of vulnerable groups: Messages that convey information about the vulnerability of specific groups or individuals to the threat. The efficacy component comprises Communication on the response efficacy, which consists of messages that convey information about the effectiveness of the recommended preventive action in averting or reducing the threat.

The components of the fear appeal message function as deliberate stimuli designed to trigger specific cognitive processes. These components demonstrate a direct correspondence with cognitive appraisals: threat elements (probability of occurrence and noxiousness) align with threat appraisal dimensions (likelihood and severity), while efficacy information maps to coping appraisal (response efficacy). This relationship is characterized by proportionality, where the intensity of message components directly influences the strength of corresponding appraisals. Thus, more vivid and powerful message elements evoke stronger cognitive responses, establishing a predictable link between external communication and internal cognitive processes. This structured alignment and proportional influence form the foundation for understanding how fear appeals shape recipients’ perceptions and subsequent behaviors (18, 38, 39).

Therefore,

*H9:* Communication on response efficacy positively affects perceived response efficacy.

*H10:* Communication on the probability of occurrence positively affects perceived likelihood.

*H11:* Communication on noxiousness positively affects perceived severity.

However, the relationship between “Communication on the susceptibility of vulnerable groups” and “Perceived susceptibility” is complex and contingent upon individual conditions, particularly one’s membership in a vulnerable group. The comparative measure of perceived susceptibility would influence this relationship. The message of susceptibility content compares the risk for vulnerable groups to the general population. At the same time, individuals are asked to assess their perceived susceptibility relative to the general population (or age group). For vulnerable group members, the impact of such communication on perceived susceptibility is likely direct and positive. The emphasis on higher risk for their group aligns with their circumstances, potentially elevating their perception of susceptibility. Conversely, for non-vulnerable individuals, the effect may be negative or neutral. By highlighting the increased risk for vulnerable groups, the message implicitly suggests lower risk for others, potentially decreasing perceived susceptibility among those who do not identify as vulnerable (18, 40).

Therefore,

*H12:* Chronic condition moderates the impact of communication on the susceptibility of vulnerable groups on perceived susceptibility.

*H12a:* Communication on the susceptibility of vulnerable groups positively affects perceived susceptibility in vulnerable groups.

*H12b:* Communication on the susceptibility of vulnerable groups negatively affects perceived susceptibility in non-vulnerable groups.

### 2.4 Moderating effects of information seeking

The moderating role of information seeking in the relationship between threat/response messages and cognitive appraisals could be elicited by examining how individuals process persuasive communications under different levels of information seeking.

The Elaboration Likelihood Model (ELM) (41, 42) is a comprehensive framework for understanding persuasion processes. The ELM has become one of the most influential social psychology and communication theories, providing insights into how people process persuasive messages and form or change their attitudes (43).

This study highlights four core tenets of ELM. First, at the ELM’s core is an elaboration continuum, which represents the degree of thought or cognitive effort an individual dedicates to processing a persuasive message. This continuum ranges from low to high elaboration, with different processes of persuasion operating at various points along this spectrum. Second, the ELM posits two primary persuasion routes: the central and peripheral. The central route, associated with high elaboration, involves scrutiny and evaluation of message arguments. In contrast, the peripheral route, linked to low elaboration, relies on simple cues or heuristics rather than effortful processing of message content. Third, where individuals fall on this elaboration continuum is largely determined by their motivation and ability to process the message. Factors such as personal relevance, need for cognition, and distractions can influence one’s motivation and ability to elaborate on a message. Fourth, the ELM proposes that the route through which an attitude is formed or changed has significant implications for the strength of that attitude. Attitudes formed through the central route (high elaboration) tend to be stronger, more enduring, more resistant to counter-persuasion, and more predictive of behavior than those formed through the peripheral route (low elaboration). This distinction in attitude strength highlights the importance of understanding whether attitude change occurs and the process through which it happens.

According to ELM, in scenarios of high information seeking, which represents a motivated state for systematic information processing (44), individuals are interested in processing messages deeply, engaging in the central route of persuasion. They pay close attention to the message components that directly correspond to cognitive appraisals. This deep processing leads to stronger cognitive appraisals because the individuals thoughtfully consider the merits of the information presented. Consequently, the impact of messages on cognitive appraisals is amplified.

Conversely, in scenarios of low information seeking, individuals are less motivated to process information deeply and are more likely to engage in peripheral route processing. They focus on superficial cues rather than the substantive content of the threat messages. As a result, the direct influence of message components on cognitive appraisals is attenuated because the individuals do not elaborate on the critical elements that align with threat appraisal dimensions (41, 45).

Therefore, information-seeking moderates the relationship between communications and cognitive appraisals by determining the route of message processing. Through central processing, high information seekers experience a stronger linkage between messages and cognitive appraisals due to deliberate and thoughtful evaluation of the message content. Low information seekers, processing messages peripherally, exhibit a weaker connection as they rely on external cues rather than engaging with the core message components. Thus,

*H13:* Information seeking moderates positively the relationship between communication messages and cognitive appraisals.

*H13a:* Information seeking moderates positively the relationship between communication on response efficacy and perceived response efficacy.

*H13b:* Information seeking moderates positively the relationship between communication on probability of occurrence and perceived likelihood.

*H13c:* Information seeking moderates positively the relationship between communication on noxiousness and perceived severity.

### 2.5 Moderating effects of information processing ease

Also, tenets of ELM can support the moderating role of information processing ease in the relationship between messages and their corresponding cognitive appraisals.

In high information processing ease scenarios, individuals find public health messages easy to understand, requiring minimal cognitive effort to comprehend the basic message content. This initial message comprehensibility enhances individuals’ ability to process information—a key determinant of ELM. In this situation, individuals are more able to process messages deeply, engaging in the central route of persuasion (42). In this path, individuals can scrutinize the message content thoroughly. They pay close attention to the substantive elements of the messages, which directly correspond to cognitive appraisals. Individuals can elaborate on the information because the messages are clear and easily digestible, leading to stronger cognitive appraisals. Consequently, the positive effects of communications on appraisals are amplified.

Conversely, in low information processing ease scenarios, individuals find the public health messages difficult to comprehend due to complexity, technical jargon, or poor presentation. This hinders their ability to process information centrally, as the increased cognitive effort reduces their capacity to engage deeply with the message content. As a result, individuals are more likely to rely on peripheral cues—such as aesthetic elements—rather than the substantive message components. The direct influence of threat and efficacy messages on cognitive appraisals is diminished because individuals cannot effectively map the message elements to their corresponding cognitive appraisals (41, 45).

Therefore, information processing ease moderates the relationship between messages and cognitive appraisals by influencing the route of message processing. High information processing ease facilitates central processing, enhancing the alignment between message components and cognitive appraisals. Low information processing ease leads to reliance on peripheral processing, weakening the direct effect of message content on cognitive appraisals. Thus,

*H14:* Information processing ease moderates positively the relationship between communication messages and cognitive appraisals.

*H14a:* Information processing ease moderates positively the relationship between communication on the response efficacy and perceived response efficacy.

*H14b:* Information processing ease moderates positively the relationship between communication on probability of occurrence and perceived likelihood.

*H14c:* Information processing ease moderates the relationship between communication on noxiousness and perceived severity positively.

### 2.6 Moderating effects of trust in government

Research suggests two distinct mechanisms influence the formation of risk perceptions from COVID-19 messages: understanding the message content (learning and comprehension of facts about threat or response efficacy) and the acceptance of this content (46, 47). The first mechanism—understanding the message—operates independently of trust in government, as empirical evidence demonstrates no significant differences in factual information acquisition between high and low-credibility sources. In other words, individuals comprehend the content of risk messages similarly, regardless of their trust level in the government as an information source.

In the high-trust scenario, when individuals trust the government (analogous to high source credibility), the acceptance mechanism operates without interference. Evidence shows the net change in agreement with advocated positions for high-credibility sources, indicating that trust enables a direct pathway between message comprehension and acceptance (46, 48). When people trust their government, understanding COVID-19 messages flows smoothly into the formation of perceptions, unimpeded by doubts about the source’s credibility. This clean cognitive pathway allows the message’s content about threat severity and likelihood to shape perceptions directly.

In the low-trust scenario, when individuals have low trust in the government (analogous to low source credibility), the acceptance mechanism is disrupted by interference. While these individuals still comprehend the messages, low trust creates a cognitive barrier between understanding and acceptance. Evidence shows no change in agreement with advocated positions for low-credibility sources (46, 48). This interference means that even though people understand the message content, their low trust in the government prevents this understanding from fully translating into perception formation. This phenomenon manifests as understanding without believing, highlighting how low trust disrupts the acceptance process while leaving comprehension intact. Thus, the relationship between messages and perception formation becomes weaker not due to failed understanding but because low trust interferes with the acceptance of the understood content. Therefore,

*H15:* Trust in government positively moderates the relationship between communication messages and cognitive appraisals.

*H15a:* Trust in government positively moderates the relationship between the communication on response efficacy and perceived response efficacy.

*H15b:* Trust in government positively moderates the relationship between communication on probability of occurrence and perceived likelihood.

*H15c:* Trust in government positively moderates the relationship between communication on noxiousness and perceived severity.

**Figure 1:**
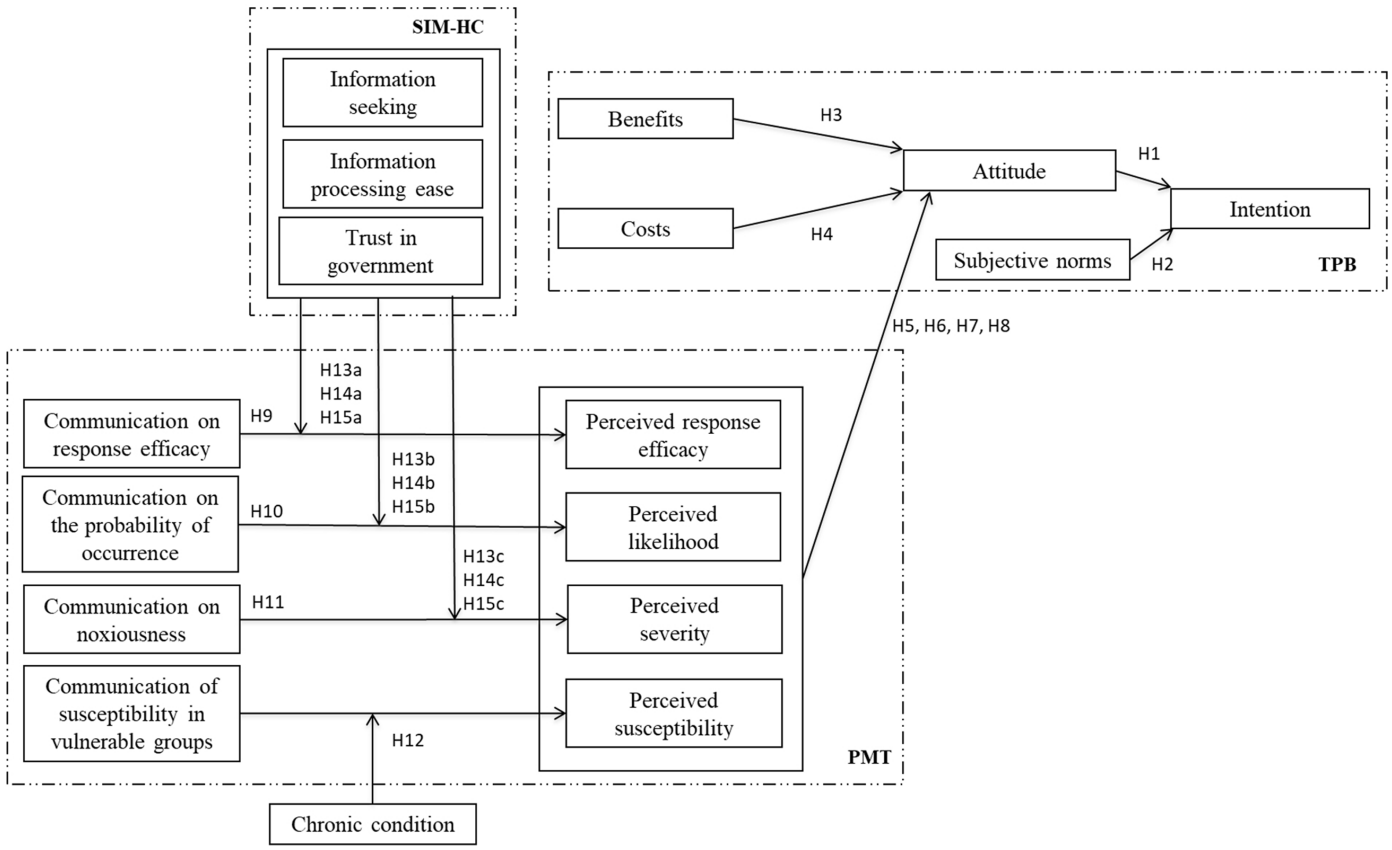
Research model.

Considering the above, Figure 1 summarizes the research model showing the contributions of each of the source theories.

## 3 Materials and methods

The present study was correlational in nature and employed a quantitative approach with a cross-sectional design. Data were gathered through a questionnaire-based survey and analyzed using the partial least squares (PLS) technique. While correlational in design, the study aimed to explain relationships between variables as proposed in the research model.

### 3.1 Measurement

The instrument was developed by using diverse tools from different authors. Sources of information were evaluated through *Probability of occurrence, Response efficacy depictions, Magnitude of noxiousness* and *Susceptibility*, which were evaluated by the tools reported by Demirtaş-Madran (38). Benefits and barriers were evaluated through *Perceived benefits* and *Perceived barriers*, for which the present study used the tool designed by Cheng and Ng (49). Risk beliefs was analyzed through different constructs. First, *Perceived severity*, which was evaluated through the tool developed by Yueng et al. and Zhang, et al. (50, 51). The *Perceived likelihood* was analyzed by using the instrument designed by Weinstein et al. (52). *Response efficacy* was evaluated by the tool reported by Witte, Cameron (53). *Perceived susceptibility* was evaluated by the tool reported by Trifiletti, Shamloo (32). About the TPB constructs, *Attitude* was evaluated through the tool designed by Park and Oh (54), *Subjective norms* were evaluated by through the instrument reported by Li, Liu (55) and *Intention* was evaluated by the tool developed by Park and Trifiletti (32, 54).

For improving content validity, the scales were adapted to the COVID-19 context. Annex presents the scales’ constituent measurement items, response formats, and sources. The researchers released a pilot test before obtaining the full-scale sample, collecting 50 preliminary observations. The analysis of internal consistency, reliability, and validity shows satisfactory indicators. Hence, minor changes were necessary for the complete sample collection.

Following literature recommendations (56), several procedures were implemented to mitigate the potential effects of common method bias (CMB). These procedures included separating the measures of the predictor from the independent variables to avoid mental connections. Additionally, various scale types (Likert, semantic differential, rating) were employed alongside diverse anchor labels (e.g., agreement, frequency, likely options) to prevent respondents from falling into a response pattern. Furthermore, the questions are presented in a manner that intersperses items from different variables throughout the questionnaire to reduce the effects of proximity.

### 3.2 Data collection

Data was collected using Amazon’s Mechanical Turk (MTurk) platform, targeting the adult American population. MTurk is particularly suitable for studies requiring large samples without specialized expertise (57). Recent research has shown that implementing certain practices can maintain the reliability of MTurk-based surveys (58, 59). Specifically, the review of Mortensen and Hughes (60) of health research found MTurk to be as efficient and reliable as traditional methods for data collection.

The researchers followed recent recommendations (57, 58, 61) to ensure data quality by implementing several measures. MTurk settings were adjusted to include only US residents with a high task completion rate (at least 98%) and a minimum of 500 completed tasks. SurveyMonkey settings prevented multiple responses from the same IP address. The questionnaire introduction emphasized the importance of attentive responses and the scientific nature of the study. Participants were informed about anonymous analysis to reduce social desirability bias and that the responses would be scrutinized, warning of rejecting invalid responses. Three attention check questions and a captcha verification were incorporated to deter bots. Compensation was set following US minimum wage laws for standard respondents.

The researchers created the survey using SurveyMonkey and made it accessible to MTurk workers. Data collection occurred between November 5th and December 10th 2023. After completing the survey, the researchers extracted the data from SurveyMonkey and compensated participants through MTurk.

### 3.3 Ethical approval

Following the ethical principles put forth in the Belmont Report, the participants gave their written informed consent before answering the questionnaire. In the informed consent, they were informed about the project, the absence of risks in answering and the privacy with which their data would be handled. The project was approved by the institutional review board of the Pontificia Universidad Católica del Perú (PUCP) (010-2023-CEI-CCSSHHyAA/PUCP).

### 3.4 Data analysis procedure

PLS is an appropriate method for examining complex relationships, handling non-normal data, and it is less demanding regarding sample size, particularly when considering multigroup analysis (61). The software used was SmartPLS 4.1.0.6.

The sample size was determined using the inverse square root method proposed by Kock and Hadaya (62). This method is particularly appropriate for the analysis as it accounts for the unique characteristics of PLS-SEM modeling. The calculated sample size requirement is 275 participants, using this approach with a minimum path coefficient of 0.15, a significance level of 0.05, and a statistical power of 80%.

Moderating effects were evaluated following the Multigroup Analysis (MGA) technique (63). The MGA method is developed specifically for the PLS-SEM context to test moderating effects by examining whether model relationships differ significantly across predefined groups. Conceptually, this aligns with moderation theory, where a third variable affects the relationship between two variables. Rather than using interaction terms, MGA directly tests whether path coefficients vary between groups formed based on the moderator variable values. The statistical significance of path differences between groups provides evidence of the moderating effect (64).

In the case of H12, the groups were divided according to the dichotomous answers of the participants regarding their health condition. For the rest of the hypotheses, following previous methodological studies, MGA focuses on extreme groups to detect differentiated effects (65). Therefore, the sample was divided into three segments based on the moderator variable: Upper, median, and lower groups, where the intermediate group corresponds to values in the median of the variable (66). The multigroup analysis was performed with the upper and lower groups.

The researchers’ choice to use extreme groups rather than a simple median split is grounded in established methodological literature. DeCoster, Iselin (65) explain that focusing “on the extreme ends of the distribution, researchers increase the differences found within their samples, which enhances the observed effects.” This approach enhances statistical power by increasing their ability to detect meaningful differences in path coefficients. It creates clearer group differentiation by excluding cases in the “gray area” near the median. This methodological strategy maximizes their ability to detect moderating effects in the PLS-SEM context.

## 4 Data analysis and results

### 4.1 Sample profile

Table 1 provides a comprehensive sample description encompassing essential demographic, socioeconomic, and health-related characteristics. These descriptors collectively represent the participants’ backgrounds and experiences pertinent to the study.

**Table 1:** Characteristics of the participants.

Characteristics	*N*	%
Sex		
Male	213	66.6
Female	105	32.8
Prefer not to answer	2	0.6
Age		
21–29	94	29.4
30–39	134	41.9
40–49	50	15.6
50–59	26	8.1
60 or older	16	5.0
Annual income		
Less than $15,000	26	8.1
From $15,000 to $29,999	43	13.4
From $30,000 to $49,999	95	29.7
From $50,000 to $74,999	90	28.1
From $75,000 to $99,999	51	15.9
From $100,000 to $150,000	11	3.4
More than $150,000	4	1.3
Last year of studies approved		
High school degree	36	11.3
Some college	23	7.2
Associate degree	15	4.7
Bachelor’s degree	197	61.6
Graduate degree	49	15.3
Economic dependents		
0	75	23.4
1	40	12.5
2	82	25.6
3	76	23.8
4	37	11.6
5	7	2.2
More than 5	3	0.9
Positive in a COVID-19 diagnostic test		
Yes	191	59.7
No	129	40.3
Prefer not to answer	0	0
Developed a severe form of COVID-19		
Yes	128	40.0
No	192	60.0
Prefer not to answer	0	0
Relatives or friends who developed a severe form of COVID-19		
Yes	200	62.5
No	119	37.2
Prefer not to answer	1	0.3
Chronic illnesses		
Yes	151	47.2
No	168	52.5
Prefer not to answer	1	0.3
Number of doses of the COVID-19 vaccine		
0	41	12.8
1	13	4.1
2	127	39.7
3	97	30.3
4	33	10.3
More than 4	9	2.8

### 4.2 Measurement model evaluation

The reliability, convergent validity, and discriminant validity were analyzed considering the cut points recommended by the literature (61). Table 2 shows that Cronbach’s alpha values were greater than 0.7 or close to that threshold, and composite reliability indicators were superior to 0.7, showing that scales had internal consistency. Also, AVE values were greater than 0.5, and the ranges of the factor loadings were over 0.7, which showed convergent validity. Table 3 shows that HTMT values were lower than 0.85 or close to that cutoff, indicating adequate discriminant validity.

**Table 2:** Composite reliability (CR), Cronbach’s alpha, average variance extracted (AVE), and range of factor loadings.

Original variable	Alias	*α*	CR	AVE	*λ* range
Attitude	Attitude	0.95	0.96	0.87	0.898–0.955
Benefits	Benefit	0.90	0.94	0.83	0.893–0.931
Chronic Condition Status	ChronicCond				
Communication on noxiousness	CommNox	0.81	0.91	0.84	0.902–0.930
Communication on response efficacy	CommRespEff				
Communication on the probability of occurrence	CommProbOccur	0.66	0.85	0.74	0.859–0.867
Communication on the susceptibility of vulnerable groups	CommSusceptVuln	0.72	0.88	0.78	0.862–0.905
Costs	Cost	0.91	0.87	0.69	0.724–0.997
Information processing ease	InfoProcEase				
Information seeking	InfoSeek				
Intention	Intention	0.93	0.96	0.88	0.929–0.954
Perceived likelihood	PercLike	0.87	0.92	0.80	0.862–0.922
Perceived response efficacy	PercRespEff	0.92	0.95	0.86	0.916–0.940
Perceived severity	PercSev	0.93	0.95	0.82	0.852–0.927
Perceived susceptibility	PercSuscept	0.93	0.96	0.88	0.935–0.949
Relevance	Relevance				
Severe Illness Development	SevereIllnessDev				
Subjective norms	SubNorm	0.93	0.96	0.88	0.940–0.942
Trust in government	TrustGov				

*α*, Cronbach’s Alpha; CR, Composite Reliability; AVE, Average Variance Extracted; *λ* range, Range of factor loadings.

**Table 3:** Heterotrait-Monotrait ratio of correlations (HTMT).

Construct	1	2	3	4	5	6	7	8	9	10	11	12	13	14
Attitude														
Benefit	0.59													
CommNox	0.06	0.25												
CommProbOccur	0.05	0.24	0.65											
CommRespEff	0.15	0.15	0.46	0.49										
CommSusceptVuln	0.03	0.12	0.60	0.60	0.44									
Cost	0.08	0.23	0.03	0.09	0.10	0.20								
Intention	0.87	0.61	0.05	0.05	0.11	0.02	0.06							
PercLike	0.65	0.51	0.09	0.13	0.04	0.08	0.21	0.71						
PercRespEff	0.84	0.66	0.05	0.13	0.09	0.07	0.08	0.86	0.68					
PercSev	0.47	0.45	0.11	0.09	0.12	0.07	0.16	0.54	0.64	0.58				
PercSuscept	0.45	0.31	0.07	0.09	0.10	0.23	0.32	0.55	0.73	0.58	0.54			
Relevance	0.60	0.41	0.02	0.08	0.11	0.03	0.11	0.72	0.60	0.58	0.44	0.49		
SevereIllnessDev	0.10	0.10	0.13	0.08	0.22	0.22	0.38	0.19	0.41	0.17	0.33	0.52	0.24	
SubNorm	0.78	0.60	0.05	0.08	0.03	0.06	0.06	0.84	0.69	0.88	0.60	0.55	0.61	0.18

Harman’s single-factor test assessed potential common method bias (CMB). Table 3 shows the analysis, which revealed that the single unrotated factor extracted accounted for 39.5% of the total variance, below the commonly accepted threshold of 50%. This result suggests that CMB is unlikely to be a significant concern in this study.

Considering the satisfactory measurement model results, the following section examines direct effects in the entire sample to evaluate hypotheses H1 through H11. Subsequently, a multigroup analysis assesses the moderation effects proposed in hypotheses H12–H15.

### 4.3 Hypothesis testing—direct effects

Before assessing the structural relationship, Variance Inflation Factors (VIFs) were calculated to evaluate potential multicollinearity issues. The analysis showed that all VIF values were below the cutoff of 5. The highest observed VIF was 2.38. These results indicate that multicollinearity was not a significant concern in the analysis.

Table 4 shows empirical support for six of the 11 hypotheses tested. Although the hypotheses linking the communication components and their respective appraisals are rejected at the level of the complete sample, as will be shown subsequently, these hypotheses are supported when analyzed in the different sample subgroups proposed in this study.

**Table 4:** Direct effects in the complete sample.

Relationship	Coeff.	*p*-value	Hypothesis evaluation
Attitude → Intention	0.527	0.000	H1: Supported
SubNorm → Intention	0.400	0.000	H2: Supported
Benefit → Attitude	0.095	0.050	H3: Supported
Cost → Attitude	−0.047	0.229	H4: Rejected
PercRespEff → Attitude	0.644	0.000	H5: Supported
PercLike → Attitude	0.235	0.000	H6: Supported
PercSev → Attitude	−0.035	0.433	H7: Rejected
PercSuscept → Attitude	−0.075	0.176	H8: Rejected
CommNox → PercSev	0.121	0.073	H9: Rejected
CommProbOccur → PercLike	0.096	0.111	H10: Rejected
CommRespEff → PercRespEff	0.041	0.487	H11: Rejected
Relevance → PercLike	0.494	0.000	Control variable
Relevance → PercRespEff	0.543	0.000	Control variable
Relevance → PercSev	0.375	0.000	Control variable
SevereIllnessDev → PercLike	0.548	0.000	Control variable
SevereIllnessDev → PercRespEff	0.093	0.244	Control variable
SevereIllnessDev → PercSev	0.500	0.000	Control variable
SevereIllnessDev → PercSuscept	0.979	0.000	Control variable

### 4.4 Hypothesis testing—moderation effects

Previous to multigroup analysis, measurement invariance across each pair of groups should be established through the Measurement Invariance of Composite Models (MICOM) procedure (67). Compositional invariance is established if the original correlation is greater than or equal to the 5% quantile of the empirical distribution of the permutation correlations for each construct. In the analysis, most of the quantiles are greater than 90% across all group comparisons and constructs, and the minimum quantile observed was 10%, above the generally accepted threshold. Therefore, measurement invariance is established.

According to the analysis procedure, an MGA was conducted for the four pairs of groups. Table 5 shows empirical support for hypotheses H12, H13, and H15. The results support partially H14. As mentioned, the hypotheses linking the communication components and their respective appraisals are supported in the upper groups of information-seeking and trust in government.

**Table 5:** Results of multigroup analysis and hypotheses evaluation.

	Path	ChronicCond-YES		ChronicCond-NO		Between groups		Hypothesis evaluation
		Coeff.	*p*-value	Coeff.	*p*-value	Difference	*p*-value	
A	CommSusceptVuln → PercSuscept	0.19	0.053	−0.27	0.000	0.46	0.000	H12: Supported

	Path	InfoSeek-HIGH		InfoSeek-LOW		Between groups		Hypothesis evaluation
		Coeff.	*p*-value	Coeff.	*p*-value	Difference	*p*-value	
B	CommRespEff → PercRespEff	0.26	0.000	−0.03	0.502	0.29	0.021	H13a: Supported
	CommProbOccur → PercLike	0.26	0.002	−0.12	0.195	0.38	0.002	H13b: Supported
	CommNox → PercSev	0.18	0.058	−0.07	0.585	0.25	0.047	H13c: Supported

	Path	InfoProcEase-HIGH		InfoProcEase-LOW		Between groups		Hypothesis evaluation
		Coeff.	*p*-value	Coeff.	*p*-value	Difference	*p*-value	
C	CommRespEff → PercRespEff	0.15	0.065	−0.09	0.347	0.24	0.029	H14a: Supported
	CommProbOccur → PercLike	0.09	0.459	0.10	0.369	−0.01	0.508	H14b: Rejected
	CommNox → PercSev	0.03	0.811	0.22	0.071	−0.19	0.884	H14c: Rejected

	Path	TrustGov-HIGH		TrustGov-LOW		Between groups		Hypothesis evaluation
		Coeff.	*p*-value	Coeff.	*p*-value	Difference	*p*-value	
D	CommRespEff → PercRespEff	0.38	0.000	−0.05	0.631	0.43	0.001	H15a: Supported
	CommProbOccur → PercLike	0.30	0.001	−0.19	0.025	0.49	0.000	H15b: Supported
	CommNox → PercSev	0.27	0.011	−0.08	0.342	0.35	0.007	H15c: Supported

## 5 Discussion

The empirical findings largely support the proposed theoretical framework while revealing important nuances in how communication dimensions influence vaccination intentions. This discussion examines five key areas: traditional TPB constructs, risk perception formation, message component effectiveness, the critical role of moderating communication factors, and the moderating role of chronic conditions.

First, the results largely support the TPB framework in the vaccination context, aligning with previous findings (68–70). For example, Dou et al. (68) found that Chinese people with stronger subjective norms have more intention to receive vaccines, while Hayashi et al. (69) determined that attitude is a significant predictor of vaccination intention in a sample of US residents. Moreover, Servidio et al. (70), who studied Italian cancer patients, found that both attitude and subjective norms were significant predictors for vaccine intention. However, the non-significant relationship between perceived costs and attitude diverges from some prior research. This finding aligns with studies in Israel (28) and Ethiopia (71) but contrasts with others (72, 73), suggesting that contextual factors such as vaccine accessibility and infrastructure may moderate this relationship.

Second, the results partially support attitude formation from risk literature, where clearly perceived response efficacy and perceived likelihood are important and sustained predictors. However, the non-significant effects of perceived severity and susceptibility on attitudes (H7, H8) diverge from pandemic-onset studies that found strong relationships (74). This temporal pattern aligns with threat perception literature in two ways. First, the significant time elapsed since the initial pandemic onset likely reduced perceived threat immediacy, diminishing both severity and susceptibility perceptions (75). Second, longitudinal studies demonstrate a pattern where severity perceptions strongly influence vaccination intentions early in health crises but wane over time (76). This temporal degradation of risk perceptions suggests that threat appraisal mechanisms may operate differently in established versus emerging health crises, pointing to the need for dynamic communication strategies across crisis lifecycles.

Third, the non-significant direct effects of message components on appraisal formation (H9–H11) in the general model highlight a key finding: health communication effectiveness operates through conditional rather than universal pathways. This suggests that traditional uniform messaging approaches may be insufficient for achieving broad public health objectives. Instead, the significant effects that emerge only under specific moderating conditions indicate the need for more nuanced communication frameworks that account for audience heterogeneity. This finding extends previous experimental studies by showing how real-world message effectiveness depends on receiver characteristics in ways that may be masked by aggregate analyses (24, 25).

Fourth, the analysis revealed nuanced patterns of message effectiveness through three key moderating variables: (a) Information-seeking behavior showed comprehensive moderation effects, with all three hypotheses (H13a–c) supported. High information seekers showed stronger alignment between message components and perceptions, consistent with findings on active information pursuit in vaccination decisions (77, 78); (b) Information processing capacity showed selective moderation effects, with only H14a supported while H14b–c were rejected. This pattern likely reflects the differential complexity of message components. For example, vaccine efficacy information requires higher processing capacity than basic likeability or severity messages. This finding extends previous research linking processing to vaccination intention (79) by showing how processing capacity interacts specifically with message complexity; (c) Government trust emerged as a crucial moderator, with H15a–c supported, reinforcing its role in public health emergency communication. This finding aligns with studies demonstrating trust’s impact on initial vaccination (80, 81) and booster acceptance (82). The selective nature of trust moderation suggests that certain message components may be more dependent on source credibility than others. These moderation effects collectively indicate that message effectiveness operates through complex pathways shaped by receiver, message, and source characteristics.

Fifth, chronic condition moderation (H12) analysis revealed differential effects of susceptibility messages between groups. This divergent pattern reflects how personal vulnerability status shapes message reception and risk perception formation. Individuals with chronic conditions with elevated COVID-19 severity risk (83–85) showed positive associations between susceptibility messages and perceived susceptibility. Conversely, those without chronic conditions demonstrated negative associations, suggesting that vulnerability-focused messages may reduce perceived susceptibility among non-vulnerable populations.

The significant between-group difference indicates that susceptibility message effectiveness depends critically on the recipient’s health status. This finding extends beyond simple health status effects to demonstrate how pre-existing conditions influence the processing and internalization of health risk messages. The pattern suggests that vulnerability-focused communication strategies may need to balance addressing high-risk populations while avoiding unintended effects on general population risk perceptions.

## 6 Implications

The study advances theoretical understanding in three key areas. First, it extends the Theory of Planned Behavior and health models by integrating communication dimensions as moderators, providing a more nuanced framework for understanding how communication shapes health behaviors. First, this framework transcends traditional TPB applications—a robust social-cognitive model that explains behavior through attitudes, subjective norms, and perceived behavioral control—by revealing how informational environments dynamically interact with core social-cognitive determinants by conceptualizing communication dimensions as distal antecedents (e.g., messages) and critical moderators (e.g., trust) in the vaccination decision process. Additionally, this study goes beyond TPB, incorporating coping and threat appraisals, creating a more comprehensive theoretical architecture delineated for health crises.

Second, the research contributes to health communication theory by revealing the temporal dynamics of risk perceptions. The findings show how threat appraisal mechanisms evolve from crisis onset to maintenance phase, suggesting theoretical models must account for these temporal shifts when explaining preventive behaviors. Third, the study enriches our understanding of message processing by demonstrating how individual characteristics (information seeking, processing capacity, trust) create distinct pathways for message effectiveness. This advances theoretical work on communication disparities by showing how receiver characteristics systematically modify message impact.

This research collectively provides a multidimensional framework, stressing communicational issues, for understanding vaccine hesitancy that integrates communication processes, cognitive-affective responses, and social influences. This integrated approach underscores the complex interplay between messages, differences in information processing, information seeking, trust, and chronic conditions, creating a more comprehensive theoretical foundation for addressing vaccine hesitancy and advancing our understanding of the dynamic, multifaceted nature of vaccination decision-making during public health emergencies.

The findings suggest three key considerations for public health managers’ vaccination campaign design. First, communication strategies should evolve strategically across crisis phases, transitioning from severity-focused messaging in early crisis periods to efficacy-focused content during maintenance phases. This temporal adaptation maintains vaccination intentions as public risk perceptions decline over time. For example, during initial outbreak phases, the emphasis should be on establishing threat salience through statistical evidence regarding virus transmission rates, exponential spread patterns, and clinical severity outcomes. Conversely, messaging should pivot toward vaccination efficacy metrics as the pandemic matures, highlighting case reduction statistics, evidence of reduced severity if infected, and information regarding minimal adverse effects from widespread immunization programs.

The findings suggest three key considerations for vaccination campaign design for public health managers. First, communication strategies could evolve across crisis phases, shifting from severity-focused messaging early in a crisis to efficacy-focused content during maintenance periods. This temporal adaptation can help maintain vaccination intentions as public risk perceptions decline.

Second, the results indicate that uniform messaging approaches may be insufficient. Public health managers should segment their audience based on information-seeking behavior, processing capacity, and trust levels, tailoring message complexity and delivery channels accordingly. For instance, providing detailed efficacy data to high information seekers while using simplified messaging for those with lower processing capacity. The delivery channels should match audience information-processing capabilities. High information seekers benefit from data-rich channels like interactive web portals and detailed scientific briefings that enable in-depth exploration. Those with lower processing capacity are better served through visual-focused channels like infographics, short videos, and community health worker interactions that present simplified content. This channel differentiation, supported by communication research, enhances message effectiveness by aligning with audience cognitive preferences and information consumption patterns.

Third, the differential effects of vulnerability messaging between chronic and non-chronic condition groups suggest the need for targeted communication strategies. While vulnerability-focused messages effectively motivate high-risk groups, they may paradoxically reduce perceived risk among the general population. This implies that public health campaigns should carefully balance addressing vulnerable populations while maintaining general public engagement. For example, campaigns might develop parallel messaging tracks—one emphasizing specific risk factors and protection strategies for individuals with chronic conditions distributed through specialist healthcare providers, and another focusing on community protection and social responsibility narratives for the general public through mass media channels—thereby maintaining engagement across population segments without inadvertently creating a false sense of security among those without chronic conditions.

For communication practitioners, the findings emphasize the importance of source credibility in message effectiveness. The strong moderating effect of government trust suggests that rebuilding institutional credibility should be a priority, particularly for ongoing vaccination campaigns. Additionally, practitioners should consider how message complexity interacts with processing capacity, ensuring technical information is presented in accessible formats while maintaining accuracy.

These implications collectively suggest that effective vaccination communication requires a more nuanced, audience-centric approach that accounts for temporal dynamics, individual characteristics, and institutional trust levels. This represents a shift from traditional uniform messaging toward more sophisticated, segmented communication strategies.

## 7 Limitations and suggestions for future studies

The study faces two methodological limitations. First, the cross-sectional design prevents establishing causal relationships between communication dimensions and vaccination intentions, as temporal precedence cannot be confirmed. This is particularly relevant given the study’s focus on the effects of evolving communication. A longitudinal study could contribute to understanding the temporal evolution of risk perceptions.

Third, the study’s focus on the U.S. context limits its generalizability to countries with different healthcare systems, cultural values, and institutional trust levels. Communication effectiveness may operate differently in contexts with varying levels of healthcare accessibility and public health infrastructure.

These limitations suggest the need for longitudinal designs, more diverse sampling methods, and cross-cultural validation in future research.

## Data Availability

The raw data supporting the conclusions of this article will be made available by the authors without undue reservation.
