# Supplementary material for: Integrating communication dimensions into health models: understanding COVID-19 vaccination through multigroup analysis
Source: Front Public Health. 2025 Jun 12;13:1609127. doi: 10.3389/fpubh.2025.1609127 (PMC12198192; doi:10.3389/fpubh.2025.1609127)
Supplement: Supplementary file 1 [file Table_1.pdf]

Annex- Items of the scale

**Construct - Source - Item wording**	
**Sources of information – (38)**	
Probability of occurrence [1]	
During the COVID-19 pandemic, the government informed the population that…	
PO1	… without a COVID-19 shot, someone's likelihood of developing severe illness was…
PO2	… with no COVID-19 shot, someone's chances of having serious symptoms were…
Response efficacy depictions [2]	
During the COVID-19 pandemic, the government informed the population that…	
RED1	… the degree to which COVID-19 vaccination could reduce the presence of serious symptoms was…
Magnitude of noxiousness [3]	
During the COVID-19 pandemic, the government informed the population that…	
MN1	… if someone did not get a COVID-19 shot, they could develop severe illness
MN2	… if someone did not get a COVID-19 shot, they could have serious symptoms
Susceptibility [4]	
During the COVID-19 pandemic, the government informed the population that…	

SUS1	… the likelihood of specific vulnerable groups (e.g., the elderly or people with diabetes) developing more severe illness from COVID-19 compared to the general population was…
SUS2	… the possibility of certain vulnerable groups (e.g., the elderly or people with diabetes) having more serious symptoms of COVID-19 compared to the general population was…
**Benefits and barriers – (49)**	
Perceived benefits [5]	
In the event of a new rise of COVID-19 infections, if I get vaccinated…	
PBE1 *	…I will be less vulnerable to COVID-19 infection.
PBE2 *	…it is less likely that I will contract COVID-19 in a public area.
PBE3 *	…I will become less anxious about contracting COVID-19.
Perceived barriers [5]	
In the event of a new rise of COVID-19 infections, if I get vaccinated…	
PBA1 *	…it will be inconvenient for me.
PBA2 *	…I will have to break my usual life habits.
PBA3 *	…my daily schedule will be disrupted.
**Risk beliefs**	
Perceived severity – (50, 51) [3]	
In the event of a new rise of COVID-19 infections, if I don't get vaccinated…	
PSE1	…my health would be seriously endangered.

PSE2	…my entire life would change negatively.
PSE3	…it could make me extremely sick.
PSE4	…I could possibly die.
Perceived likelihood – (52)[6]	
In the event of a new rise of COVID-19 infections, if I don't get vaccinated…	
PL1	…I think my chances of developing severe illness from COVID-19 would be…
PL2 *	…I think my likelihood of developing severe illness from COVID-19 is…
PL3	…I would expect to develop severe illness from COVID-19.
Response efficacy – (53) [3]	
In the event of a new rise of COVID-19 infections, how much do you agree or disagree with the following three statements?	
RE1	1) Getting a new dose of the vaccine could work in preventing severe illness from COVID-19.
RE2	2) Getting a new dose of the vaccine could be effective in preventing serious symptoms from COVID-19.
RE3	3) If I get vaccinated, I would be less likely to get a severe illness from COVID-19.
Perceived susceptibility –(32) [2]	
In the event of a new rise of COVID-19 infections…	
PSU1	…compared to most people my age, my risk of developing severe illness from COVID-19 is…

PSU2	…compared to most people my age, the likelihood of me getting serious symptoms from COVID-19 is…
PSU3	…compared to most people, the possibility of me developing severe illness from COVID-19 is…
**Theory of Planned Behavior**	
Attitude – (54) [7]	
A1 *	COVID-19 vaccination is… (beneficial - harmful)
A2 *	COVID-19 vaccination is… (necessary - unnecessary)
A3 *	COVID-19 vaccination is… (useful - useless)
A4 *	COVID-19 vaccination is… (important - unimportant)
Subjective norms – (55) [3]	
In the event of a new rise of COVID-19 infections, how much do you agree or disagree with the following three statements?	
NS1	1) The people who are important to me would think that I should get vaccinated against COVID-19.
NS2	2) The people who have influence in my life would think that I should get vaccinated against COVID-19.
NS3	3) The people whose opinion matters to me would think that I should get vaccinated against COVID-19.
Intention – (32, 54) [8]	
In the event of a new rise of COVID-19 infections...?	
I1 *	I would intend to get the COVID-19 vaccine.

I2	How willing would you be to get the COVID-19 vaccine?
I3	Would you intend to get a vaccination for COVID-19?
**Relevance** [9]	
Rel	How relevant is the COVID-19 issue for you currently?

[*] Reversed items

[1] Scale: Very unlikely … Very likely // Very small … Very large

[2] Scale: Extremely low … Extremely high

[3] Scale: Strongly disagree … Strongly agree

[4] Scale: Equally likely … Extremely more likely // Equally possible … Extremely more possible

[5] Scale: Strongly agree … Strongly disagree

[6] Scale: Almost zero … Almost certain // Very likely … Very unlikely // Strongly disagree … Strongly agree

[7] Scale: Very beneficial … Very harmful // Very necessary … Very unnecessary // Very useful … Very useless // Very important … Very unimportant

[8] Scale: Strongly agree … Strongly disagree // Definitely unwilling … Definitely willing // Definitely will not … Definitely will

[9] Scale: Very irrelevant … Very relevant
